# Renal replacement therapy prior to liver transplant and inpatient mortality in patients without advanced kidney disease: A nationwide study

**DOI:** 10.1002/jgh3.13028

**Published:** 2024-01-03

**Authors:** Hassam Ali, Vishali Moond, Cameron Lawson, Deepa Budh, Ritika Ohri, Pratik Patel, Wong Yu Jun, Eduardo Rodriguez‐Zarate, Babu P. Mohan

**Affiliations:** ^1^ Department of Medicine and Gastroenterology East Carolina University/Brody School of Medicine Greenville North Carolina USA; ^2^ Department of Internal Medicine Saint Peter's University Hospital/Robert Wood Johnson Medical School New Brunswick New Jersey USA; ^3^ Department of Internal Medicine St. Barnabas Hospital/Albert Einstein College of Medicine New York New York USA; ^4^ Department of Nephrology University of Utah Health School of Medicine Salt Lake City Utah USA; ^5^ Department of Gastroenterology Mather Hospital/Hofstra University Zucker School of Medicine Port Jefferson New York USA; ^6^ Department of Gastroenterology & Hepatology Changi General Hospital, SingHealth Singapore; ^7^ Duke‐NUS Medical School Singapore; ^8^ Department of Gastroenterology & Hepatology University of Utah School of Medicine Salt Lake City Utah USA

**Keywords:** liver transplant, nationwide analysis, renal replacement therapy

## Abstract

**Background and Aim:**

The utility of renal replacement therapy (RRT) before liver transplant (LT) in patients without end‐stage renal disease (ESRD) or advanced chronic kidney disease (CKD‐IV/V) is debatable and lacks data support. We aimed to evaluate the impact of RRT on patients undergoing LT.

**Methods:**

We used the National Readmission Database (2016–2019) to identify all index hospitalizations undergoing RRT before LT (cases). A matched comparison cohort of similar hospitalizations without RRT before LT was identified (controls) after 1:1 propensity score matching for age, gender, and available comorbidities.

**Results:**

We matched 364 cases (RRT before LT) to 364 controls (LT without prior RRT). There was no statistical difference in all‐cause inpatient mortality (4.9% *vs* 3.6% *P* = 0.4). A significantly greater proportion of cases were associated with ICU admission (40.7% *vs* 17.0%, *P* < 0.001) and RRT requirement post LT (100% *vs* 17%, *P* < 0.001). There was no difference in 30‐ (hazard ratio [HR] 1.1, 0.4–2.6), 60‐ (HR 0.9, 0.4–1.8), or 90‐day (HR 0.8, 0.4–1.6) inpatient mortality between the groups. Also, 180‐day survival estimates were comparable (*P* = 0.5). The results were similar in patients with no chronic kidney disease (CKD) and CKD‐III.

**Conclusion:**

RRT prior to LT, in patients without advanced CKD or ESRD, was associated with greater instances of ICU stay and need for future RRT. Also, 30‐, 60‐, and 90‐day inpatient mortality rates were similar, and 180‐day survival estimates were comparable.

## Graphical abstract

Renal replacement therapy prior to liver transplantation in patients without advanced chronic kidney disease does not effect mortality outcomes.

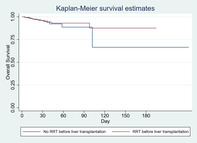

## Introduction

About 10–20% of patients with end‐stage liver disease (ESLD) present with concomitant renal dysfunction, and the prevalence of renal failure has been reported to be as high as 70% post liver transplant (LT).^1^, ^2^, ^3^ Renal dysfunction in the presence of ESLD has been associated with increased morbidity and mortality post LT.^1^, ^2^, ^3^ Renal replacement therapy (RRT), in the form of continuous renal replacement therapy (CRRT) or hemodialysis (HD), is frequently used to manage the peri‐operative course of LT patients with renal dysfunction or chronic kidney disease (CKD).

Indications for RRT in LT patients include anuria, electrolyte imbalance, hypervolemia, or vasopressor requirement in the presence of renal dysfunction.^1^, ^2^, ^3^ There is some thought among transplant surgeons that CRRT may assist with the optimization of a patient's overall clinical status prior to LT, despite not meeting explicit indications set forth by nephrologists. Therefore, in the absence of set indications, the request for RRT prior to LT breeds controversy among the treatment teams. Moreover, it is a fairly common practice to place sick patients awaiting LT on CRRT to artificially increase the model for end‐stage liver disease (MELD) score to expedite organ availability.

Currently, the clinical outcomes of pre‐LT RRT, in the absence of clear‐cut indications, are unknown due to the paucity of data. Additionally, there has been limited evidence to date on the use of RRT before LT in patients without renal dysfunction. To address this gap, we aimed to study the effects of pre‐LT RRT on post‐LT clinical outcomes. In this study, we aimed to assess the clinical outcomes of RRT performed prior to LT surgery by comparing the study population to a propensity‐score‐matched cohort who did not have pre‐LT RRT.

## Methods

### Design and data source

The present retrospective study utilized the Nationwide Readmission Database (NRD) from 2016 to 2019, which was developed by the Agency for Healthcare Research and Quality's Healthcare Cost and Utilization Project (HCUP) (HCUP‐US NIS Overview. Available from: https://www.hcup-us.ahrq.gov/nisoverview.jsp). We used the NRD because it provides recognition of index admissions and repeated hospitalizations (readmissions) for the same patients, using a unique patient linkage identification number.^4^ NRD includes data on about 18 million discharges annually in the United States from 28 states, including patient‐ and hospital‐level data. NRD 2016 and above used the International Classification of Diseases (ICD)10 coding system to store and report data. (Detailed information on NRD design and sampling methods is available at https://www.hcup-us.ahrq.gov.) All ICD‐10 codes used in the present study are given in Table SS1. Since the present study is a cross‐sectional assessment of a nationwide database, the STROBE (strengthening the reporting of observational studies in epidemiology) checklist was followed (details provided in Appendix A).

### Study population

Hospitalization cases that underwent RRT before LT (cases) were identified using the database. A matched comparison cohort of similar hospitalizations who did not undergo RRT before LT (controls) was identified from NRD after 1:1 propensity score matching using age, gender, and Elixhauser comorbidities showing good balance (Table S2). After identifying the index admission from NRD, we calculated the time to RRT and time to LT using procedure codes and included patients in the study only if the time to RRT was less than the time to LT, thereby correctly identifying patients who had RRT prior to LT.

Hospitalized patients with age <18 years, a history of malignant neoplasm, lymphomas, end‐stage renal disease (ESRD or advanced CKD stage IV/V as defined by the glomerular filtration rate [GFR] <15 ml/min for ESRD/CKD‐V and 15–29 mL/min for CKD‐IV), prior renal transplant, solid‐organ malignancies without metastasis, or paraplegia/paresis were excluded from the analysis. These conditions were deemed high‐risk conditions that could confound the analysis. In this study, “malignant neoplasm” refers to any metastatic cancer, while “solid‐organ malignancies without metastasis” denote non‐metastatic cancers in organs such as the liver, kidney, and lungs. This distinction, excluding hematologic malignancies, is crucial for accurately assessing patient eligibility and outcomes in LT. Additionally, hospitalizations during December were excluded as the individual 1‐year NRD data files do not have a corresponding month to capture 30‐day readmission.

### Outcomes

This study evaluated inpatient clinical outcomes of cases (patients with LT who underwent RRT prior to LT surgery) in index hospitalizations. Inpatient outcomes included median hospital length of stay (LOS), hospital charges, all‐cause inpatient mortality, intensive care unit (ICU) admission, and other complications (LT‐ and RRT‐related). In addition to overall assessment of outcomes for CKD stages I (GFR > 90 mL/min), II (GFR = 60–89 mL/min), and III (GFR = 30–59 mL/min), subgroup analysis was performed for CKD stage III and no CKD. Subgroup analysis for CKDI/II was not performed because of the smaller patient sample (*N* = 3).

### Statistical analysis

Statistical analysis was performed using Statistical Software for Data Science (STATA 16).

Each hospitalization was assigned a propensity score using a multivariable logistic regression, which included individual‐ (age, sex, race, and comorbidities) and hospital‐level (location, teaching status, and bed size) variables. Cases and controls were matched based on the propensity scores in a 1:1 manner within 0.01 standard deviation of the calculated score, as in prior literature.^5^, ^6^ The covariate balance was then visualized using a two‐way plot shown in Figures S1a (before the match) and S1b (after the match). A two‐sample Wilcoxon rank‐sum (Mann–Whitney) test was used for continuous variables, and outcomes were reported with interquartile ranges (IQRs). The Chi‐square test was used to compare categorical variables, and outcomes were reported as frequency (%). The measures of association were reported as odds ratios (ORs) with 95% confidence intervals (CIs). For mortality, hazard ratios (HRs) were reported with 95% CI. Kaplan–Meier curve was generated to demonstrate overall survival between cases and controls, and significance was assessed using the log‐rank test. According to the HCUP Data Use Agreement, any values below ten have been masked and reported as “<10” to ensure privacy and compliance.

## Results

### Baseline demographics

Overall, 728 patients were included in the analysis. The top primary diagnoses for LT hospitalizations, based on the ICD codes, were alcoholic cirrhosis with ascites and acute/ subacute liver failure without coma (Table S3). As ESRD/CKD‐IV cases were excluded, all study patients were either CKD‐III or below at baseline before LT.

We matched 364 hospitalizations with RRT before LT (cases) to 364 hospitalizations with no RRT before LT (controls). Adequate comorbidity balance was achieved with propensity matching between the comparison groups, as summarized in Table S2, including CKD stages (I– III), obesity, and fluid and electrolyte disorder. The median age was 51.5 years in cases (IQR 43–59) and 54 years in controls (IQR 46–61). The time to LT was longer in cases compared to controls (13.5 days [IQR 8–20] *vs* 1 day [IQR 0–8], *P* < 0.001). Private insurance was the primary payer (Table 1), and there was no difference in median household income in both cohorts (*P* = 0.11).

**Table 1:** Baseline characteristics and inpatient outcomes after propensity matching of comparison groups (ESRD & CKD‐IV excluded)

Factor	RRT before liver transplant (cases)	No RRT before liver transplant (controls)	*P*‐value
N	364	364	
Length of stay (days), median (IQR)	32 (22–46)	14 (9–30)	<0.001
Total charges (USD), median (IQR)	827 695 (574 184– 1 354 623)	467 809 (323 809– 724 291)	<0.001
Elixhauser comorbidity index (ECI) score			0.85
0	0 (0.0%)	0 (0.0%)	
1	<10 (0.3%)	<10 (0.5%)	
2	<10 (1.4%)	<10 (1.4%)	
≥3	358 (98.4%)	357 (98.1%)	
Primary payer			0.005
Medicare	63 (18.4%)	80 (23.5%)	
Medicaid	92 (26.9%)	68 (20.0%)	
Private	180 (52.6%)	192 (56.5%)	
Other	<10 (2.0%)	0 (0.0%)	
Median household income national quartile for patient ZIP code			0.11
First (0–25th)	81 (22.3%)	77 (21.2%)	
Second (26th–50th)	69 (19.0%)	92 (25.3%)	
Third (51st–75th)	107 (29.4%)	110 (30.2%)	
Fourth (76th–100th)	107 (29.4%)	85 (23.4%)	
Disposition of patient			0.012
Discharged to home or self‐care (routine discharge)	115 (31.6%)	160 (44.0%)	
Transfer to short‐term hospital	<10 (1.1%)	<10 (1.6%)	
Transfer others: includes skilled nursing facility (SNF), intermediate care facility (ICF), another type of facility	76 (20.9%)	64 (17.6%)	
Home health care (HHC)	151 (41.5%)	121 (33.2%)	
Died during hospitalization	18 (4.9%)	13 (3.6%)	0.36
Age in years at admission, median (IQR)	51.5 (43.0–59.0)	54.0 (46.0–61.0)	0.003
Age groups (years)			0.019
18–34	50 (13.7%)	30 (8.2%)	
34–49	108 (29.7%)	96 (26.4%)	
50–64	175 (48.1%)	190 (52.2%)	
65–79	31 (8.5%)	48 (13.2%)	
Mechanical ventilation	159 (43.7%)	59 (16.2%)	<0.001
Septic shock	163 (44.8%)	78 (21.4%)	<0.001
Cardiac arrest	0 (0.0%)	0 (0.0%)	‐
ICU admission	148 (40.7%)	62 (17.0%)	<0.001
Time to ICU, median (IQR)	5.0 (1.0–14.0)	4.0 (1.0–10.0)	0.20
Vasopressor requirement	87 (23.9%)	52 (14.3%)	<0.001
RRT post LT	364 (100.0%)	62 (17.0%)	<0.001
Liver transplant rejection	19 (5.2%)	22 (6.0%)	0.63
Acute kidney injury	348 (95.6%)	276 (75.8%)	<0.001
No chronic kidney disease	337 (92.58%)	337 (92.58%)	1.00
Chronic kidney disease (Stage I‐II)	<10 (0.82%)	<10 (0.27%)	0.31
Chronic kidney disease (Stage III)	24 (6.59%)	26 (7.14%)	0.76
Biliary disease (obstruction, unspecified)	28 (7.7%)	43 (11.8%)	0.061
Bile duct exploration	<10 (0.3%)	<10 (0.3%)	1.00
Liver transplant infection	0 (0.0%)	<10 (1.4%)	0.025
Upper extremity VTE	10 (2.7%)	<10 (1.4%)	0.19
Lower extremity VTE	<10 (2.2%)	11 (3.0%)	0.49
Pulmonary embolism	<10 (0.3%)	<10 (0.8%)	0.32
Portal venous thrombosis	55 (15.1%)	54 (14.8%)	0.92
Urinary tract infection	82 (22.5%)	34 (9.3%)	<0.001
Catheter‐associated urinary tract infection	<10 (0.5%)	<10 (0.3%)	0.56

ICU, intensive care unit; RRT, renal replacement therapy; VTE, venous thromboembolism.

There was no statistical difference in all‐cause inpatient mortality between cases and controls (4.9% *vs* 3.6%, *P* = 0.4). Pre‐LT RRT (cases) was associated with longer median LOS at 32 days (IQR 22–46) compared to no pre‐LT RRT (controls) at 14 days (IQR 9–30) (*P* < 0.001). Additionally, the median total hospital charges were also higher in cases ($827 695 [IQR 574 184–1 354 623] compared to controls $467 809 [IQR 323 809–724 291], *P* < 0.001). There was a greater proportion of “Discharge to Home” or “Self‐Care” in controls (44.0%) compared to cases (31.6%) (*P* = 0.01).

A significantly greater proportion of cases had a higher association with mechanical ventilation (43.7% *vs* 16.2%, OR 4.0 [95% CI: 2.8–5.7], *P* < 0.001), septic shock (44.8% *vs* 21.4%, OR 2.9 [95% CI: 2.1–4.1], *P* < 0.001), ICU admission (40.7% *vs* 17.0%, OR 3.3 [95% CI: 2.4–4.7], *P* < 0.001), vasopressor requirement (23.9% *vs* 14.3%, OR 1.9 [95% CI: 1.3–2.8], *P* < 0.001), and acute kidney injury (95.6% *vs* 75.8%, OR 6.9 [95% CI: 3.9–12.1], *P* < 0.001) compared to controls. RRT requirement post LT (during same hospitalization) was significantly greater in cases than in controls (100% *vs* 17%, *P* < 0.001).

**Figure 1:**
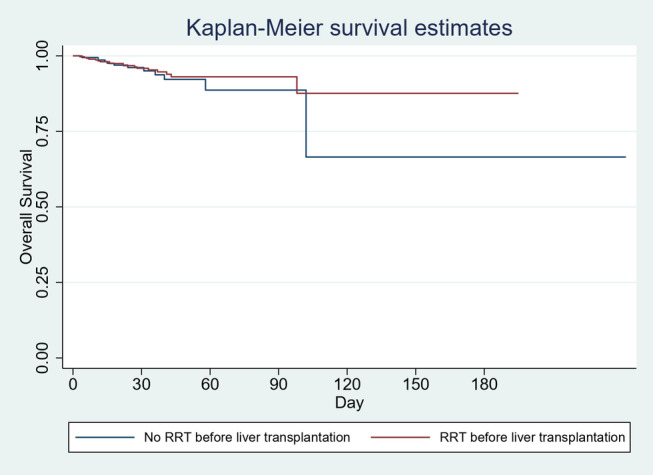
Kaplan–Meier survival estimates (overall).

**Figure 2:**
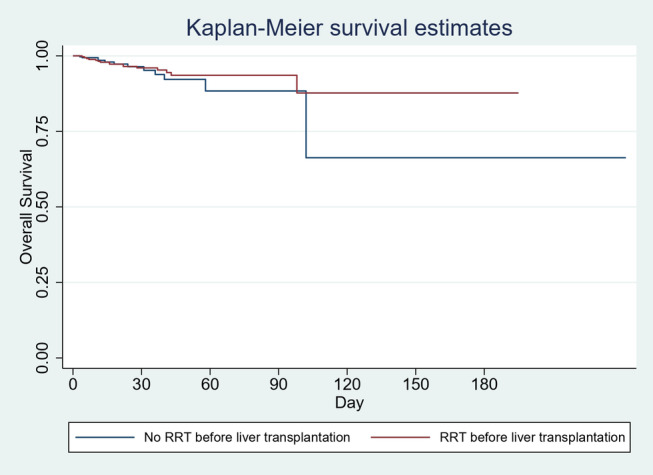
Kaplan–Meier survival estimates (for no CKD).

Cases were associated with 77% of unspecified biliary disease and biliary obstruction as compared to 11.8% in controls (*P* = 0.06). However, the association of LT infection was 0% in cases as compared to 1.4% in controls. There was no difference in the risk of inpatient mortality at 30‐ (HR 1.06 [95% CI: 0.4–2.6], *P* = 0.9), 60‐ (HR 0.85 [95% CI: 0.4–1.8], *P* = 0.7), or 90‐day intervals (HR 0.79 [95% CI: 0.4–1.6], *P* = 0.5) (Fig. 2) between the groups. Also,180‐day Kaplan–Meier survival estimates were comparable (*P* = 0.5) (Fig. 1). The results are summarized in Table 1.

### Patients with no CKD

After matching, 337 cases were compared with 337 controls. Similar results were noted for LOS and hospital charges (summarized in Table 2). The median age was 51 in cases (IQR 43–59) and 53 in controls (IQR 46–60).

**Table 2:** Subgroup analysis of matched cohorts based on underlying kidney disease

	No CKD			CKD (Stage III)		
Factor	RRT before liver transplant (cases)	No RRT before liver transplant (controls)	*P*‐value	RRT before liver transplant (cases)	No RRT before liver transplant (controls)	*P*‐value
*N*	337	337		26	24	
Length of stay (days), median (IQR)	32.0 (22.0–46.0)	14.0 (9.0, 29.0)	<0.001	30.0 (27.0–53.0)	13.5 (8.5–32.0)	0.001
Total charges (USD), median (IQR)	831 545.0 (577 667.5–1.3 × 10^6^)	450 089.0 (319 400.0–719 583.0)	<0.001	834 791.0 (567 240.0–1.3 × 10^6^)	510 463.0 (355 444.0–789 391.0)	0.020
Elixhauser comorbidity index (ECI) score			0.85			
0	<10 (0.3%)	<10 (0.6%)		0 (0.0%)	0 (0.0%)	
1	<10 (1.5%)	<10 (1.5%)		0 (0.0%)	0 (0.0%)	
2	331 (98.2%)	330 (97.9%)		0 (0.0%)	0 (0.0%)	
≥3			0.008	26 (100%)	24 (100%)	
Primary payer	57 (18.0%)	70 (22.3%)				0.43
Medicare	86 (27.2%)	65 (20.7%)		<10 (24%)	<10 (39%)	
Medicaid	166 (52.5%)	179 (57.0%)		<10 (24%)	<10 (13%)	
Private	<10 (2.2%)	<10 (0.0%)		13 (52%)	11 (48%)	
Other			0.11	0 (0.0%)	0 (0.0%)	
Median household income national quartile for patient ZIP code	76 (22.6%)	74 (22.0%)				0.89
First (0–25th)	61 (18.1%)	83 (24.6%)		<10 (19%)	<10 (13%)	
Second (26th–50th)	97 (28.8%)	99 (29.4%)		<10 (27%)	<10 (33%)	
Third (51st–75th)	103 (30.6%)	81 (24.0%)		10 (38%)	10 (42%)	
Fourth (76th–100th)			0.015	<10 (15%)	<10 (13%)	
Disposition of patient						0.61
Discharged to home or self‐care (routine discharge)	110 (32.6%)	151 (44.8%)		<10 (19%)	<10 (29%)	
Transfer to short‐term hospital	<10 (0.9%)	<10 (1.8%)		<1 (4%)	0 (0%)	
Transfer to other: includes skilled nursing facility (SNF), intermediate care facility (ICF), another type of facility	73 (21.7%)	59 (17.5%)		<10 (12%)	<10 (21%)	
Home health care (HHC)	135 (40.1%)	109 (32.3%)		15 (58%)	11 (46%)	
Died during hospitalization	16 (4.7%)	12 (3.6%)	0.44	<10 (8%)	<10 (4%)	0.60
Age in years at admission, median (IQR)	51.0 (43.0–59.0)	53.0 (46.0–60.0)	0.006	57.0 (51.0–60.0)	60.5 (50.5–65.0)	0.14
Age groups (years)			0.043			0.060
18–34	47 (13.9%)	30 (8.9%)		<10 (12%)	0 (0%)	
34–49	105 (31.2%)	90 (26.7%)		3 (12%)	5 (21%)	
50–64	156 (46.3%)	176 (52.2%)		18 (69%)	12 (50%)	
65–79	29 (8.6%)	41 (12.2%)		2 (8%)	7 (29%)	
Mechanical ventilation	153 (45.4%)	53 (15.7%)	<0.001	6 (23%)	6 (25%)	0.87
Septic shock	147 (43.6%)	70 (20.8%)	<0.001	15 (58%)	7 (29%)	0.042
Cardiac arrest	141 (41.8%)	59 (17.5%)	<0.001	7 (27%)	3 (13%)	0.20
ICU admission	4.0 (1.0, 14.0)	3.0 (1.0, 10.0)	0.16	7.5 (6.0, 23.0)	6.0 (2.5, 31.0)	0.46
Time to ICU, median (IQR)	80 (23.7%)	47 (13.9%)	0.001	6 (23%)	5 (21%)	0.85
Vasopressor requirement	153 (45.4%)	53 (15.7%)	<0.001	6 (23%)	6 (25%)	0.87
RRT	337 (100.0%)	53 (15.7%)	<0.001	26 (100%)	8 (33%)	<0.001
Liver transplant rejection	15 (4.5%)	19 (5.6%)	0.48	4 (15%)	3 (13%)	0.77
Acute kidney injury	321 (95.3%)	252 (74.8%)	<0.001	26 (100%)	21 (88%)	0.063
Biliary disease (obstruction, unspecified)	27 (8.0%)	42 (12.5%)	0.057	1 (4%)	1 (4%)	0.95
Bile duct exploration	<10 (0.3%)	<10 (0.3%)	1.00	0 (0%)	0 (0%)	
Liver transplant infection	0 (0.0%)	<10 (1.2%)	0.045	0 (0%)	1 (4%)	0.29
Upper extremity VTE	10 (3.0%)	<10 (1.2%)	0.11	0 (0%)	1 (4%)	0.29
Lower extremity VTE	<10 (2.4%)	11 (3.3%)	0.49	0 (0%)	0 (0%)	
Pulmonary embolism	<10 (0.3%)	<10 (0.9%)	0.32	0 (0%)	0 (0%)	
Portal venous thrombosis	49 (14.5%)	49 (14.5%)	1.00	6 (23%)	4 (17%)	0.57
Urinary tract infection	77 (22.8%)	32 (9.5%)	<0.001	5 (19%)	2 (8%)	0.27
Catheter‐associated urinary tract infection	<10 (0.6%)	<10 (0.3%)	0.56	0 (0%)	0 (0%)	

ICU, intensive care unit; RRT, renal replacement therapy; VTE, venous thromboembolism.

Cases were associated with greater instances of mechanical ventilation (45.4% *vs* 15.7%, *P* < 0.001), septic shock (43.6% *vs* 20.8%, *P* < 0.001), cardiac arrest (41.8% *vs* 17.5%, *P* < 0.001), vasopressor requirement (45.4% *vs* 15.7%, *P* < 0.001), AKI (95.3% *vs* 74.8%, *P* < 0.001), and requirement of RRT post LT (100% *vs* 15.7%, *P* < 0.001).

Cases were associated with 8% of biliary disease as compared to 12.5% in controls (*P* = 0.06). Association of LT infection was 0% in cases as compared to 1.2% in controls (*P* = 0.05). For patients with no underlying CKD, there was no difference in the risk of inpatient mortality at 30‐ (HR 1.2 [95% CI: 0.5–2.9], *P* = 0.74), 60‐ (HR 0.8 [95% CI: 0.4–1.8], *P* = 0.66), or 90‐day intervals (HR 0.8 [95% CI: 0.4–1.8], *P* = 0.6). Also, 180‐day Kaplan–Meier survival estimates were comparable (*P* = 0.5) (Fig. 2). The results are summarized in Table 2.

### Patients with CKD‐III

After matching, 26 cases were compared with 24 controls. The median age in cases was 57 years (IQR 51–60) and in controls 60.5 years (IQR 50.5–65). Greater association of septic shock (58% *vs* 29%, *P* = 0.04), AKI (100% *vs* 88%, *P* = 0.06), and RRT post LT (100% *vs* 33%, *P* < 0.01) was noted with cases as compared to controls. Results are summarized in Table 2.

**Figure 3:**
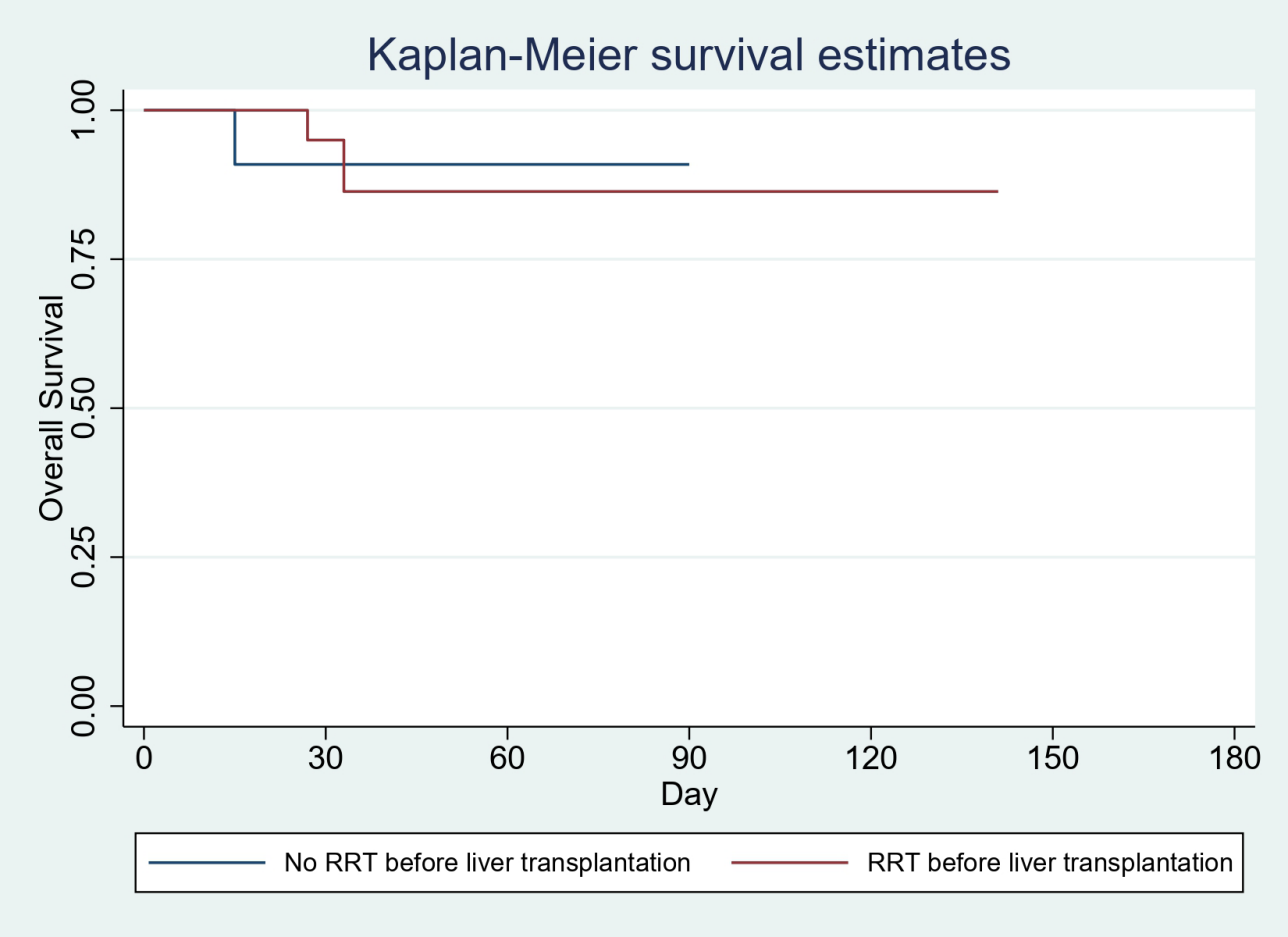
Kaplan–Meier survival estimates (for CKD‐III).

No differences were noted in the association of liver transplant rejection, infection, biliary diseases, or ICU admissions. For patients with underlying CKD stage III, there was no difference in the risk of inpatient mortality at 30‐ (HR 0.39 [95% CI: 0.1–6.30], *P* = 0.51), 60‐ (HR 0.88 [95% CI: 0.07–9.79], *P* = 0.91), or 90‐day intervals (HR 0.88 [95% CI: 0.08–9.81], *P* = 0.92). Also, 180‐day Kaplan–Meier survival estimates were comparable (*P* = 0.91) (Fig. 3). Results are summarized in Table 2.

## Discussion

Based on this propensity‐score‐matched analysis of the database representative of the U.S. population, there were no differences in the risk of 30‐, 60‐, or 90‐day inpatient mortality between patients undergoing RRT prior to LT and no RRT prior to LT. Additionally, patients who underwent RRT prior to LT were associated with significantly more instances of ICU care, AKI, and requirement of RRT after LT. Furthermore, the 180‐day survival estimates were comparable between the groups. These associations were consistent for patients with no CKD or CKD‐III at baseline. To the best of our knowledge, this is the first study of its kind evaluating the association of inpatient outcomes of performing RRT prior to LT in patients without ESRD or advanced CKD.

The practice of RRT before LT is variable across different institutions. Currently it is unclear if pre‐LT RRT, particularly when performed beyond the current established indications for RRT, could result in meaningful clinical improvement following LT. Owing to the absence of data on this topic, current management is driven by the individual patient's clinical features along with a multidisciplinary decision‐making process.^1^, ^2^, ^3^ In this study, we found that pre‐LT RRT did not confer survival benefits among transplant recipients. Our findings provide novel and supporting evidence to guide decision making on pre‐LT RRT, particularly among transplant waitlist candidates beyond the established indications for RRT.

Patients who received RRT prior to LT were associated with greater instances of ICU care, mechanical ventilation, cardiac arrest, vasopressor requirement, and septic shock. It is important to note that these results from the cross‐sectional analysis of a database only suggest association and not causation. We cannot tell whether these events led to the initiation of RRT, or RRT was the cause for these observations. A prospective or retrospective analysis of longitudinal data would be needed to assess causality. Nevertheless, these results are novel and have not been reported earlier in the literature.

In the current study, patients undergoing pre‐LT RRT, regardless of CKD stage, had a 100% requirement of continuation of RRT after LT post transplant during the same hospitalization. Additionally, there was a greater association of AKI in cases as compared to controls. Any degree of intradialytic hypotension during RRT causes ischemic injury to the kidneys, thus reducing the rate of renal recovery. In the matched controls (i.e., LT patients with no RRT prior to surgery), 15.7% of patients with normal renal function and 33% with CKD‐III required RRT. Similarly, documented diagnosis code for AKI was significantly lower in patients who did not get RRT pre‐LT.

One of the criteria for a simultaneous liver and kidney transplant is a patient with GFR < 60 mL/min (CKD‐III or worse) who is on RRT. Although the underlying patient variables in terms of CKD stages were controlled via propensity score matching, the combination of RRT and CKD‐III by themselves could have contributed toward the increased requirement of RRT in CKD‐III patients post LT. However, it is important to note that a significantly greater need for RRT was noted in the no‐CKD group as well. Therefore, it is safe to conclude that initiation of RRT before LT has contributed to the significantly greater requirement of RRT after LT, regardless of the underlying CKD stage.

The reasons for the need of RRT and the causes of AKI in the control cohort are unknown because of the lack of granular data. We did not report on the outcomes for patients with CKD‐IV or ESRD, as these patients were not our population of interest and so were excluded. In a single‐center retrospective study of adult patients with GFR < 30 mL/min who underwent LT, Safwan et al. divided the study cohort into three groups: CRRT prior to LT, intraoperative CRRT, and no CRRT. Postoperative complications rates were similar in all three groups, including long‐term renal function. However, the group that had CRRT initiated before LT showed the highest rate of postoperative RRT and a higher risk of 1‐year graft loss and mortality.^7^

In terms of liver‐related issues, biliary disease (coded as unspecified obstruction) and liver transplant infection were less associated with patients who received pre‐LT RRT. Although the differences were not significant, the findings are interesting. These observations were noted in patients with no CKD. Further studies are needed to assess this observation with granular patient‐level data. There was no difference in graft rejection between the study groups. Associations of other complications such as venous thromboembolism, pulmonary embolism, and catheter‐associated infections were comparable for both cohorts.

Strengths of this study are primarily related to the novelty of the findings in this topic derived from a large inpatient database. After identifying the index admission from NRD, patients with RRT before LT were accurately identified using the time to RRT and time to LT. Additionally, NRD provides data from single hospitalization; therefore, all the study patients underwent LT during the same inpatient stay. Therefore, the reported associations between RRT and LT are robust. However, limitations exist primarily in not being able to assess causality, which is inherent to any cross‐sectional database analysis. Future studies from longitudinally collected data are warranted to establish our findings and assess potential causality of the outcomes to RRT *versus* no RRT prior to LT. We lacked individual patient‐level data, lab values, MELD scores, and data on severity of underlying cirrhosis, which might have influenced the post‐transplant survival. Nevertheless, this study adds valuable information to the current literature on the role of RRT prior to LT.

## Conclusion

In conclusion, based on the analysis of national U.S. readmission database, RRT prior to LT was associated with greater instances of ICU stay, vasopressor requirement, septic shock, use of mechanical ventilation, AKI, and need for RRT post LT. Also, the 30‐, 60‐, and 90‐day inpatient mortality were similar between RRT and no RRT prior to LT groups, along with comparable 180‐day survival estimates. The results were consistent for CKD‐III and no CKD. Further longitudinal studies are warranted to establish causality.

## Ethics approval

Review board approval and patient consent were not required, as this is an analysis of a retrospectively maintained, de‐identified national inpatient sample database.

## Supporting information


**Table S1.** List of ICD‐10 codes used in the present study.
**Table S2.** Comorbidity balance after propensity matching of comparison groups.
**Table S3.** Top five primary diagnoses for LT hospitalizations.
**Figure S1.** Propensity‐score‐matched densities.
**Appendix A.** STROBE statement—Checklist of items that should be included in reports of cross‐sectional studies.
