# Supplementary material for: Renal replacement therapy prior to liver transplant and inpatient mortality in patients without advanced kidney disease: A nationwide study
Source: JGH Open. 2024 Jan 3;8(1):e13028. doi: 10.1002/jgh3.13028 (PMC10805480; doi:10.1002/jgh3.13028)
Supplement: Supplementary file 1 — Table S1. List of ICD‐10 codes used in the present study. Table S2. Comorbidity balance after propensity matching of comparison groups. Table S3. Top five primary diagnoses for LT hospitalizations. Figure S1. Propensity‐score‐matched densities. Appendix A. STROBE statement—Checklist of items that should be included in reports of cross‐sectional studies. [file JGH3-8-e13028-s001.docx]

**Supplementary materials**

**Supplementary materials legend:**

Supplementary table 1: List of ICD-10 codes utilized in the present study

Supplementary Table 2: Comorbidity balance after propensity matching of comparison groups

Supplementary Figure 1A/B, Propensity matched densities

Supplementary Table 3: Top five primary diagnosis for LT hospitalizations

Appendix A: STROBE Statement—Checklist of items that should be included in reports of *cross-sectional studies*

Supplementary table 1: List of ICD-10 codes utilized in the present study

| Variables | ICD-10 codes |
| --- | --- |
| Liver transplantation | 0FY00Z0, 0FY00Z1, 0FY00Z2* |
| RRT (CRRT & Hemodialysis) | 5A1D00Z, 5A1D60Z, 5A1D90Z, 5A1D80Z, 5A1D70Z* |
| Biliary disease (obstruction or cyst) | K831, K835, K838 |
| Bile duct exploration | 0FC90ZZ* |
| Bile duct anastomosis repair | 0FQ98ZZ, 0FQ97ZZ* |
| Liver transplant infection | T8643 |
| Upper extremity venous thromboembolism | I82601, I82602, I82603, I82609, I82611, I82612, I82613, I82619, I82621, I82622, I82623, I82629 |
| Lower extremity venous thromboembolism | I82401, I82402, I82403, I82409, I82411, I82412, I82413, I82419, I82421, I82422, I82423, I82429, I82431, I82432, I82433, I82439, I82441, I82442, I82443, I82449, I82491, I82492, I82493, I82499, I824Y1, I824Y2, I824Y3, I82499, I824Y1, I824Y2, I824Y3, I824Y9, I824Z1, I824Z2, I824Z3, I824Z9 |
| Pulmonary embolism | I2601, I2602, I2609, I2690, I2692, I2699 |
| Portal venous thrombosis | I81 |
| UTI | N390, N3000, N3001 |
| CAUTI | T83511A |
| AKI | N170, N171, N172, N178, N179 |
| ESRD, Previous HD use | N184, N186 |
| CKD stage I | N181 |
| CKD stage II | N182 |
| CKD stage III | N183 |
| History of kidney transplant | Z940, Z4822  0TY00Z0, 0TY00Z1, 0TY00Z2, 0TY10Z0, 0TY10Z1, 0TY10Z2* |
| Mechanical ventilation | 5A1945Z, 5A1935Z, 5A1955Z* |
| Septic shock | R652, A419, R6520 |
| ICU admission | 5A1955Z, 5A1935Z, 5A1945Z, 03HY03Z, 03HY33Z, 03HY43Z, 04HY03Z, 04HY33Z, 04HY43Z, 4A133B1, 4A13XB1, 4A14XB1* |
| Vasopressor use | 3E030XZ, 3E033XZ, 3E040XZ, 3E043XZ, 3E050XZ, 3E053XZ, 3E060XZ, 3E063XZ* |
| Liver transplant rejection | T8641, T8642 |
| Congestive Heart Failure | I09.9, I11.0, I13.0, I13.2, I25.5, I42.0, I42.5–I42.9,  I43, I50, P29.0 |
| Cardiac Arrhythmia | I44.1–I44.3, I45.6, I45.9, I47–I49, R00.0, R00.1,  R00.8, T82.1, Z45.0, Z95.0 |
| Valvular Disease | A52.0, I05–I08, I09.1, I09.8, I34–I39, Q23.0–Q23.3,  Z95.2–Z95.4 |
| Pulmonary Circulation Disorders | I26, I27, I28.0, I28.8, I28.9 |
| Peripheral Vascular Disorders | I70, I71, I73.1, I73.8, I73.9, I77.1, I79.0, I79.2, K55.1,  K55.8, K55.9, Z95.8, Z95.9 |
| Hypertension without Complications | I10 |
| Diabetes without Complications | E10.0, E10.1, E10.9, E11.0, E11.1, E11.9, E12.0,  E12.1, E12.9, E13.0, E13.1, E13.9, E14.0, E14.1,  E14.9 |
| Diabetes with Complications | E10.2–E10.8, E11.2–E11.8, E12.2–E12.8,  E13.2–E13.8, E14.2–E14.8 |
| Hypothyroidism | E00–E03, E89.0 |
| Liver Disease | B18, I85, I86.4, I98.2, K70, K71.1, K71.3–K71.5,  K71.7, K72–K74, K76.0, K76.2–K76.9, Z94.4 |
| Peptic Ulcer Disease excluding Bleeding | K25.7, K25.9, K26.7, K26.9, K27.7, K27.9, K28.7,  K28.9 |
| Metastatic Cancer | C77–C80 |
| Solid Tumor without Metastasis | C00-C26, C30-C34, C37-C41, C43, C45-C58, C60-  C76, C97 |
| Coagulopathy | D65–D68, D69.1, D69.3–D69.6 |
| Obesity | E66 |
| Protein calorie malnutrition | E40–E46, R63.4, R64 |
| Fluid and Electrolyte Disorders | E22.2, E86, E87 |
| Psychoses | F20, F22–F25, F28, F29, F30.2, F31.2, F31.5 |
| Depression | F20.4, F31.3–F31.5, F32, F33, F34.1, F41.2, F43.2 |

*Procedure codes

Supplementary Table 2: Comorbidity balance after propensity matching of comparison groups

| Factor | RRT before Liver transplant (cases) | No RRT before Liver transplant (controls) | p-value |
| --- | --- | --- | --- |
| N | 364 | 364 |  |
| Congestive heart failure | 15 (4.1%) | 15 (4.1%) | 1.00 |
| Cardiac arrhythmias | 66 (18.1%) | 66 (18.1%) | 1.00 |
| Valvular disease | 2 (0.5%) | 2 (0.5%) | 1.00 |
| Pulmonary circulation | 11 (3.0%) | 11 (3.0%) | 1.00 |
| Peripheral vascular disease | 6 (1.6%) | 6 (1.6%) | 1.00 |
| Uncomplicated hypertension | 69 (19.0%) | 69 (19.0%) | 1.00 |
| Chronic pulmonary diseases | 12 (3.3%) | 12 (3.3%) | 1.00 |
| Uncomplicated diabetes | 3 (0.8%) | 3 (0.8%) | 1.00 |
| Complicated diabetes | 55 (15.1%) | 55 (15.1%) | 1.00 |
| Hypothyroidism | 14 (3.8%) | 14 (3.8%) | 1.00 |
| Chronic kidney disease (Stage I-III) | 27 (7.4%) | 27 (7.4%) | 1.00 |
| Peptic ulcer disease excluding bleeding | 2 (0.5%) | 2 (0.5%) | 1.00 |
| Rheumatoid arthritis/Collagen Vascular disorder | 5 (1.4%) | 5 (1.4%) | 1.00 |
| Coagulopathy | 331 (90.9%) | 331 (90.9%) | 1.00 |
| Obesity | 46 (12.6%) | 46 (12.6%) | 1.00 |
| Weight loss | 224 (61.5%) | 224 (61.5%) | 1.00 |
| Fluid and electrolyte disorder | 348 (95.6%) | 348 (95.6%) | 1.00 |
| Blood loss anemia | 9 (2.5%) | 6 (1.6%) | 0.43 |
| Deficiency anemia | 18 (4.9%) | 18 (4.9%) | 1.00 |
| Alcohol abuse | 186 (51.1%) | 186 (51.1%) | 1.00 |
| Drug abuse | 7 (1.9%) | 7 (1.9%) | 1.00 |
| Psychoses | 1 (0.3%) | 1 (0.3%) | 1.00 |
| Depression | 42 (11.5%) | 42 (11.5%) | 1.00 |
| Complicated hypertension | 42 (11.5%) | 42 (11.5%) | 1.00 |

Supplementary Figure 1A/B, Propensity matched densities

A


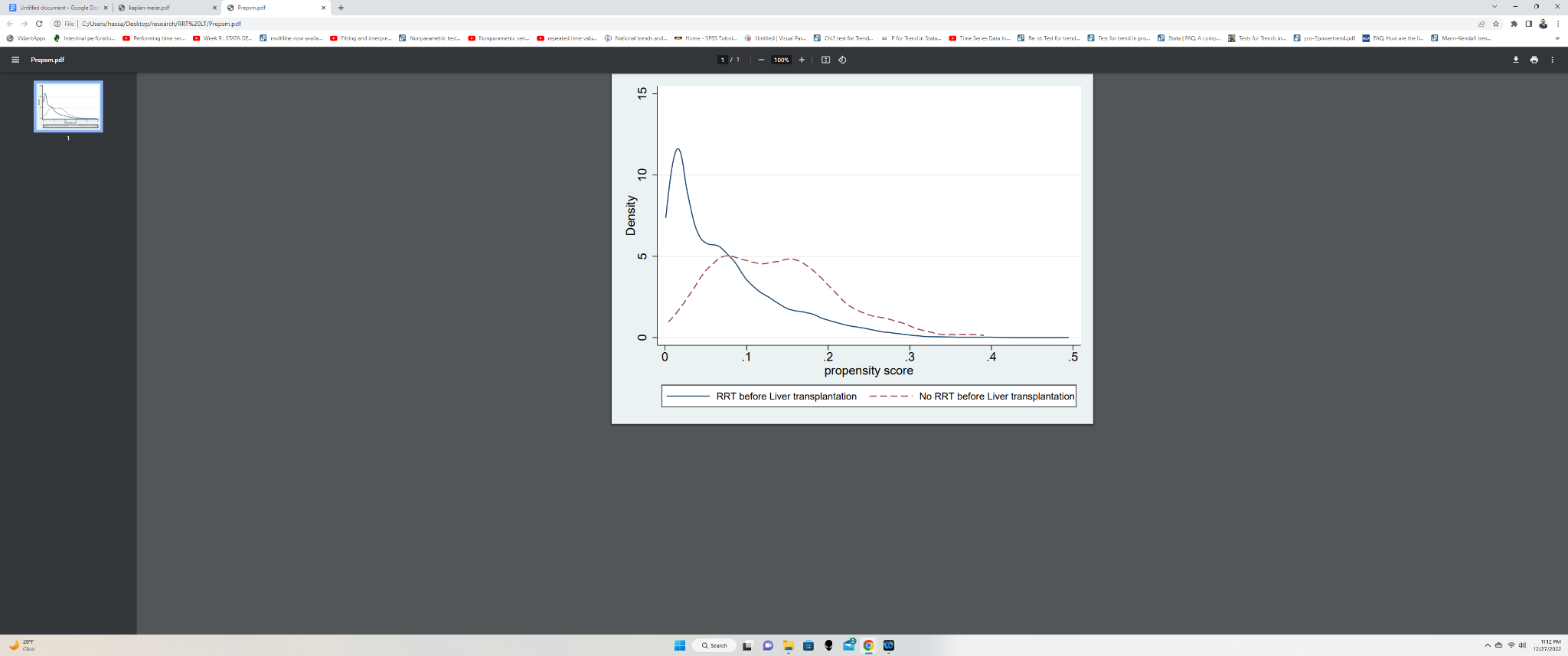


B


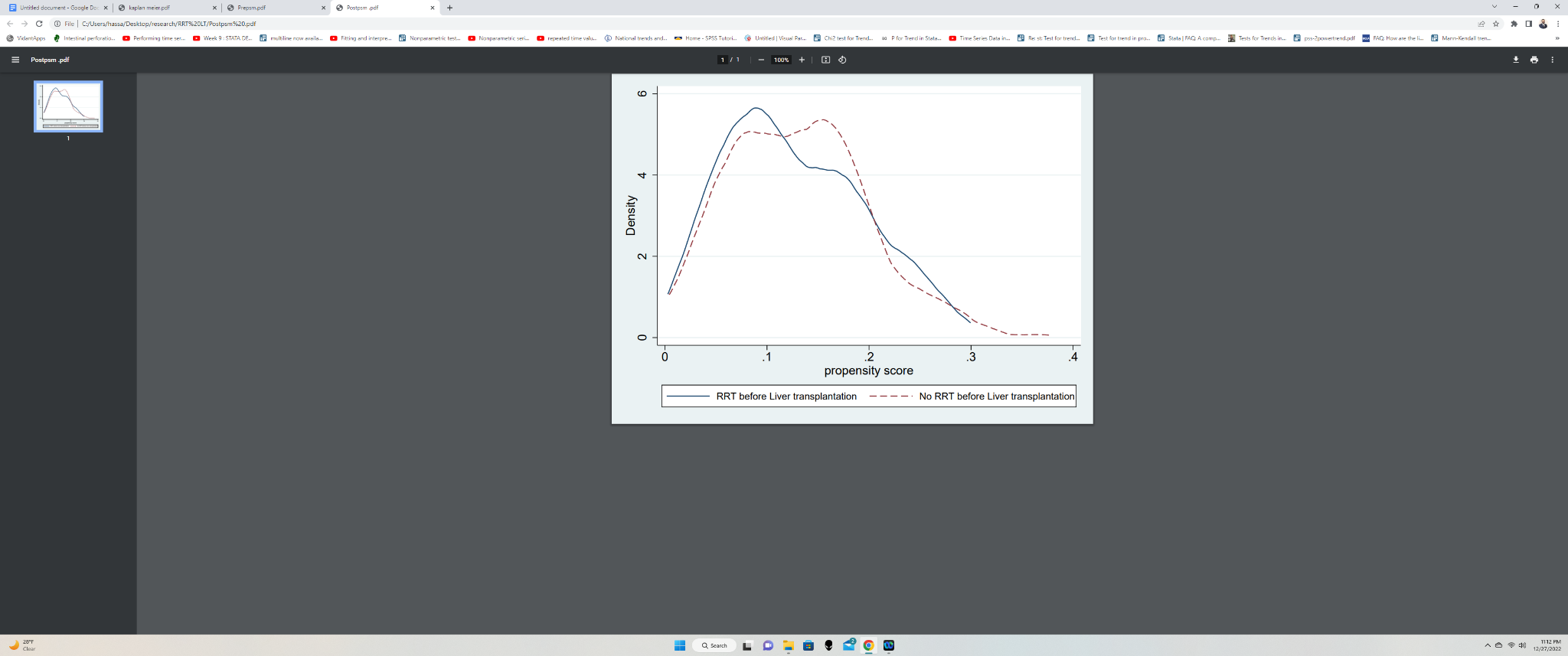


Supplementary Table 3: Top five primary diagnosis for LT hospitalizations

| Primary Diagnosis | Frequency (N%) |
| --- | --- |
| Alcoholic cirrhosis of liver with ascites | 25.1% |
| Acute and subacute hepatic failure without coma | 9.4% |
| Other cirrhosis of liver | 7.55% |
| Hepatic failure, unspecified without coma | 6.31% |
| Alcoholic hepatic failure without coma | 6.31% |

Appendix A: STROBE Statement—Checklist of items that should be included in reports of ***cross-sectional studies***

|  | Item No | Recommendation |
| --- | --- | --- |
| **Title and abstract** | 1 | (*a*) Indicate the study’s design with a commonly used term in the title or the abstract - done |
|  |  | (*b*) Provide in the abstract an informative and balanced summary of what was done and what was found - done |
| Introduction | | |
| Background/rationale | 2 | Explain the scientific background and rationale for the investigation being reported - done |
| Objectives | 3 | State specific objectives, including any prespecified hypotheses - done |
| Methods | | |
| Study design | 4 | Present key elements of study design early in the paper - done |
| Setting | 5 | Describe the setting, locations, and relevant dates, including periods of recruitment, exposure, follow-up, and data collection - done |
| Participants | 6 | (*a*) Give the eligibility criteria, and the sources and methods of selection of participants - done |
| Variables | 7 | Clearly define all outcomes, exposures, predictors, potential confounders, and effect modifiers. Give diagnostic criteria, if applicable - done |
| Data sources/ measurement | 8* | For each variable of interest, give sources of data and details of methods of assessment (measurement). Describe comparability of assessment methods if there is more than one group - done |
| Bias | 9 | Describe any efforts to address potential sources of bias - done |
| Study size | 10 | Explain how the study size was arrived at - NA |
| Quantitative variables | 11 | Explain how quantitative variables were handled in the analyses. If applicable, describe which groupings were chosen and why - done |
| Statistical methods | 12 | (*a*) Describe all statistical methods, including those used to control for confounding - done |
|  |  | (*b*) Describe any methods used to examine subgroups and interactions - done |
|  |  | (*c*) Explain how missing data were addressed - NA |
|  |  | (*d*) If applicable, describe analytical methods taking account of sampling strategy - done |
|  |  | (*e*) Describe any sensitivity analyses - done |
| Results | | |
| Participants | 13* | (a) Report numbers of individuals at each stage of study—eg numbers potentially eligible, examined for eligibility, confirmed eligible, included in the study, completing follow-up, and analysed - done |
|  |  | (b) Give reasons for non-participation at each stage- done |
|  |  | (c) Consider use of a flow diagram- NA |
| Descriptive data | 14* | (a) Give characteristics of study participants (eg demographic, clinical, social) and information on exposures and potential confounders- done |
|  |  | (b) Indicate number of participants with missing data for each variable of interest- done |
| Outcome data | 15 | Report numbers of outcome events or summary measures- done |
| Main results | 16 | (*a*) Give unadjusted estimates and, if applicable, confounder-adjusted estimates and their precision (eg, 95% confidence interval). Make clear which confounders were adjusted for and why they were included- done |
|  |  | (*b*) Report category boundaries when continuous variables were categorized- done |
|  |  | (*c*) If relevant, consider translating estimates of relative risk into absolute risk for a meaningful time period- done |
| Other analyses | 17 | Report other analyses done—eg analyses of subgroups and interactions, and sensitivity analyses- done (subgroup analysis performed based on CKD stage) |
| Discussion | | |
| Key results | 18 | Summarise key results with reference to study objectives- done |
| Limitations | 19 | Discuss limitations of the study, taking into account sources of potential bias or imprecision. Discuss both direction and magnitude of any potential bias- done |
| Interpretation | 20 | Give a cautious overall interpretation of results considering objectives, limitations, multiplicity of analyses, results from similar studies, and other relevant evidence - done |
| Generalisability | 21 | Discuss the generalisability (external validity) of the study results - done |
| Other information | | |
| Funding | 22 | Give the source of funding and the role of the funders for the present study and, if applicable, for the original study on which the present article is based – proved in title page |

STROBE Initiative is available at www.strobe-statement.org.
